# Initial Bacterial Adhesion on Different Yttria-Stabilized Tetragonal Zirconia Implant Surfaces *in Vitro*

**DOI:** 10.3390/ma6125659

**Published:** 2013-12-04

**Authors:** Lamprini Karygianni, Andrea Jähnig, Stefanie Schienle, Falk Bernsmann, Erik Adolfsson, Ralf J. Kohal, Jérôme Chevalier, Elmar Hellwig, Ali Al-Ahmad

**Affiliations:** 1 Division of Restorative Dentistry and Periodontology, Department of Dental Medicine, Oral and Maxillofacial Surgery, Medical Center, University of Freiburg, Hugstetter Str. 55, Freiburg 79106, Germany; E-Mails: lamprini.karygianni@uniklinik-freiburg.de (L.K.); andrea.saw@web.de (A.J.); stefanieschienle@web.de (S.S.); elmar.hellwig@uniklinik-freiburg.de (E.H.); 2 NTTF Coatings GmbH, Maarweg 32, Rheinbreitbach 53619, Germany; E-Mail: falk.bernsmann@nttf-coatings.de; 3 Ceramic Materials, Swerea IVF, Mölndal SE-431 22, Sweden; E-Mail: Erik.Adolfsson@swerea.se; 4 Division of Prosthodontics, Department of Dental Medicine, Oral and Maxillofacial Surgery, Medical Center, University of Freiburg, Hugstetter Str. 55, Freiburg 79106, Germany; E-Mail: ralf.kohal@uniklinik-freiburg.de; 5 University of Lyon, INSA-Lyon, MATEIS UMR CNRS 5510, 20 Avenue Albert Einstien, Villeurbanne 69621, France; E-Mail: jeromechevalier@me.com

**Keywords:** antimicrobial surface, initial bacteria adhesion, live/dead staining, yttria-stabilized zirconia, zirconia ceramics

## Abstract

Bacterial adhesion to implant biomaterials constitutes a virulence factor leading to biofilm formation, infection and treatment failure. The aim of this study was to examine the initial bacterial adhesion on different implant materials *in vitro*. Four implant biomaterials were incubated with *Enterococcus faecalis*, *Staphylococcus aureus* and *Candida albicans* for 2 h: 3 mol % yttria-stabilized tetragonal zirconia polycrystal surface (B1a), B1a with zirconium oxide (ZrO_2_) coating (B2a), B1a with zirconia-based composite coating (B1b) and B1a with zirconia-based composite and ZrO_2_ coatings (B2b). Bovine enamel slabs (BES) served as control. The adherent microorganisms were quantified and visualized using scanning electron microscopy (SEM); DAPI and live/dead staining. The lowest bacterial count of *E. faecalis* was detected on BES and the highest on B1a. The fewest vital *C. albicans* strains (42.22%) were detected on B2a surfaces, while most *E. faecalis* and *S. aureus* strains (approximately 80%) were vital overall. Compared to BES; coated and uncoated zirconia substrata exhibited no anti-adhesive properties. Further improvement of the material surface characteristics is essential.

## 1. Introduction

Due to its considerably higher strength with corresponding fracture resistance, yttria-stabilized tetragonal zirconia (Y-TZP) has been recognized as a favorable ceramic biomaterial the last two decades [1]. Y-TZP has been widely used in various biomedical applications, especially for the construction of femoral heads for total hip replacements and dental implants [2,3]. Nevertheless, Y-TZP and 3 mol% yttria-stabilized tetragonal zirconia (3Y-TZP) failed to remain stable over time. More specifically, in 2001 a total of 400 femoral heads had to be removed shortly after their implantation as a result of accelerated ageing in two implant batches fabricated with a new furnace technology [4]. Their predilection to low temperature degradation (LTD) in the presence of water, known as ageing, constitutes their Achilles heel [5,6]. A mechanism named tetragonal to monoclinic transformation (t-m transformation), responsible for converting the metastable tetragonal grains to monoclinic, seems to cause this disadvantageous feature [7]. Nowadays, the development of innovative structural ceramic biomaterials that can withstand high pressure over time remains a challenge in the field of implant research.

Oral biofilms are dynamic microbial structures that can adhere to various surfaces in the oral cavity [8,9]. These specialized bacterial communities can tolerate the harsh environmental conditions associated with gingival tissues, tooth and implant surfaces [10,11,12]. The discovery and comprehension of the biofilm formation mechanisms have been in focus of the biofilm research community over the past few years [13]. Oral biofilms have been proved to play a causative role in biofilm-mediated diseases such as caries, periodontis and periimplantitis. This fact has triggered the interest for the invention of material surfaces with antibacterial properties [14,15]. As far as dental materials are concerned, possible discrepancies in the initial rates of bacterial colonization on different implant surfaces need to be investigated [16]. Material surface characteristics such as average surface roughness (Sa), root mean square surface roughness (Sq), ten-point average roughness (Sz), skewness (Ssk), summit density (Sds), developed area ratio (Sdr) and texture aspect ratio (Str) can influence the initial microbial colonization rate as well as the strength and structural properties of biofilms [17]. Moreover, the counteraction of oral biofilms with other mechanical, physical and chemical factors relating to material substrata, microorganisms and adsorbed macromolecules complicate their methodological examination [18].

The aim of this study was to investigate the initial bacterial adhesion on four novel implant material surfaces *in vitro* utilizing bovine enamel slabs (BES) as a control. The implant material surfaces utilized for the examination of the initial bacterial colonization *in vitro* were: 3 mol% yttria-stabilized tetragonal zirconia polycrystal surface (B1a), 3 mol% yttria-stabilized tetragonal zirconia polycrystal surface with zirconium oxide (ZrO_2_) coating (B2a), 3 mol% yttria-stabilized tetragonal zirconia polycrystal surface with zirconia-based composite coating (B1b) and 3 mol% yttria-stabilized tetragonal zirconia polycrystal surface with zirconia-based composite and ZrO_2_ coatings (B2b). The state-of-the-art implant material Y-TZP served as a benchmark. The composite coating is a material designed to withstand ageing. Therefore, it is a potential material for future implants and as such, its behavior with respect to microbial adhesion is of great interest. As the employed plasma-deposited ZrO_2_ coating is very hydrophilic, a good ingrowth of coated implants is expected. A possible influence of the ZrO_2_-coating on microbial adhesion was investigated in the present work.

Due to the structural similarity to human enamel, BES were considered to be appropriate for this purpose [19]. Staining with 4',6-diamidino-2-phenylindole (DAPI) aided the quantitative analysis of all adherent microorganisms, while their vitality was determined using live/dead staining. Additionally, the initial adherent microorganisms as well as the implant material surfaces were visualized by scanning electron microscopy (SEM).

## 2. Results and Discussion

**Figure 1:**
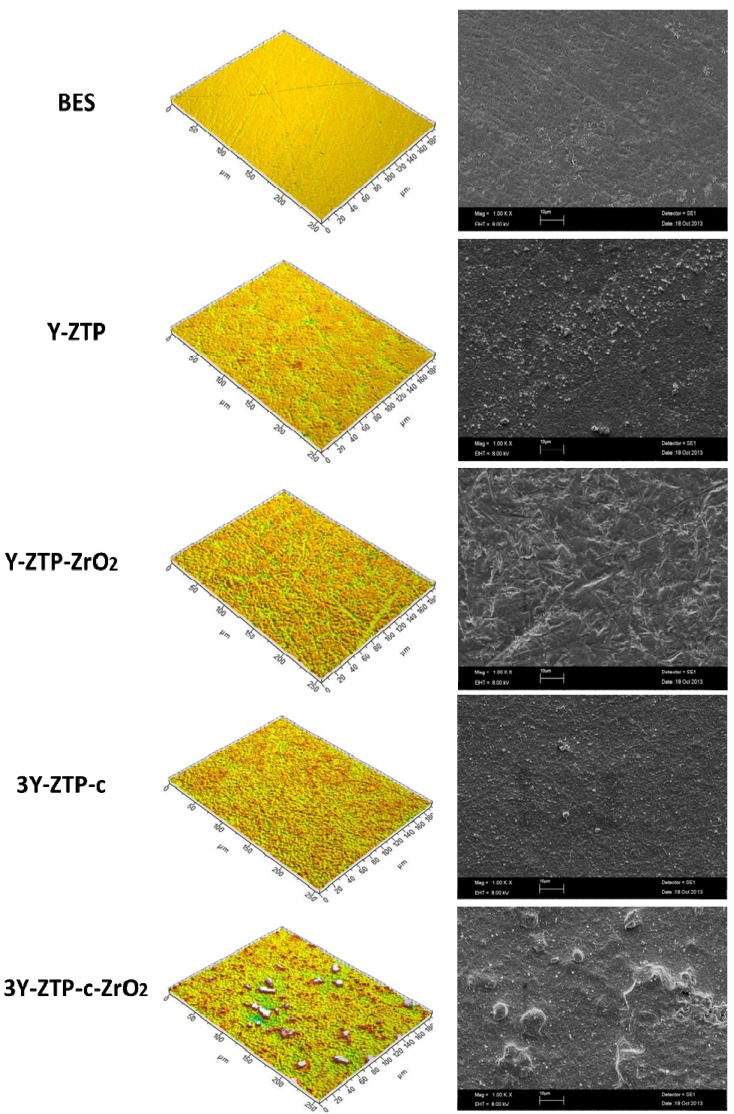
Scanning electron microscopic (SEM) as well as interferometric images of the four different implant material surfaces and the control (BES). SEM magnification: 1000 fold; scale bar: 10 μm.

Table 1 and Figure 1 summarize the five different implant material surfaces (diameter, 15 mm; surface area, 176.62 mm^2^; height, 1.5 mm) used for the examination of the initial bacterial colonization *in vitro*: 3 mol% yttria-stabilized tetragonal zirconia polycrystal surface (B1a), 3 mol% yttria-stabilized tetragonal zirconia polycrystal surface with zirconium oxide (ZrO_2_) coating (B2a), 3 mol% yttria-stabilized tetragonal zirconia polycrystal surface with zirconia-based composite coating (B1b) and 3 mol% yttria-stabilized tetragonal zirconia polycrystal surface with zirconia-based composite and ZrO_2_ coatings (B2b). All experimental ceramic materials were fabricated at Swerea IVF (Mölndal, Sweden) and at NTTF Coatings GmbH (Rheinbreitbach, Germany).

**Table 1:** The sample names and brief description of the tested implant material surfaces.

Samples	Material Description	Coating
BES	Bovine enamel slabs	No
B1a	3 mol% yttria-stabilized tetragonal zirconia polycrystal surface	No
B2a	3 mol% yttria-stabilized tetragonal zirconia polycrystal surface	zirconium oxide (ZrO_2_) coating
B1b	3 mol% yttria-stabilized tetragonal zirconia polycrystal surface	zirconia-based composite coating
B2b	3 mol% yttria-stabilized tetragonal zirconia polycrystal surface	zirconia-based composite and zirconium oxide (ZrO_2_) coatings

After the acquirement of the implant material surfaces, control surfaces were prepared as well. For the obtainment of the control specimens, the buccal surfaces of 140 bovine incisors of 2 year old cattle were removed and modified into cylindrical enamel slabs (diameter, 5 mm; surface area, 19.63 mm^2^; height, 1.5 mm). The IDEXX Laboratories bovine spongiform encephalopathy (BSE) diagnostic kit (Ludwigsburg, Germany) confirmed their BSE-free status. Wet grinding with abrasive paper (400 to 4000 grit) was then used to polish the enamel surfaces of all BES samples. The protocol for disinfection of the enamel plates involved dislodgement of the superficial smear layer by ultrasonication in NaOCl (3%) for 3 min, air drying, and ultrasonication in 70% ethanol for another 3 min. The disinfected samples were ultrasonicated twice again in double-distilled water for 10 min and, subsequently kept in distilled water for 24 h to hydrate.

The visualization of the implant surface morphology was performed by SEM. Moreover, various amplitude-, hybrid- and spatial parameters concerning the tested biomaterial surfaces were measured by interferometry (Table 2). In Table 2, the surface roughness parameters Sa, Sq and Sz increase with each coating step. After coating no polishing or any other further treatment was conducted.

**Table 2:** The main surface characteristics of the tested implant material surfaces.

Materials	Surface characteristics						
	Amplitude Parameters				Hybrid Parameters		Spatial Parameters
	Average surface roughness (Sa)	Root mean square surface roughness (Sq)	Ten-point average roughness (Sz)	Skewness (Ssk)	Summit density (Sds)	Developed area ratio (Sdr)	Texture aspect ratio (Str)
**BES (control)**	0.041 μm	0.056 μm	0.98 μm	−0.72	0.141/μm²	0.48%	0.220
**B1a**	0.119 μm	0.162 μm	2.44 μm	−0.64	0.108/μm²	2.23%	0.734
**B2a**	0.199 μm	0.255 μm	3.58 μm	−0.685	0.100/μm²	5.98%	0.841
**B1b**	0.252 μm	0.384 μm	4.54 μm	1.43	0.110/μm²	7.20%	0.799
**B2b**	0.259 μm	0.408 μm	4.97 μm	1.97	0.111/μm²	7.44%	0.353

**Figure 2:**
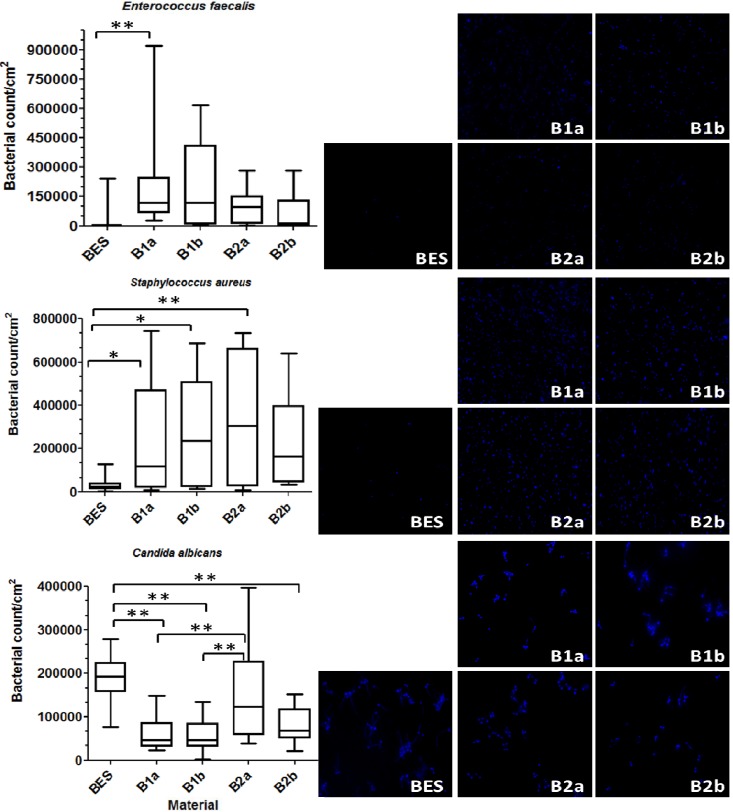
Boxplots depicting the bacterial count of *Enterococcus faecalis*, *Staphylococcus aureus* and *Candida albicans* on the different implant material surfaces and the control after an exposure time of 2 h. The medians, whiskers, and max/min outliers are displayed. The images on the right show the initial bacterial adhesion of the tested microorganisms on the implant material surfaces after 2 h examined by DAPI staining and followed by fluorescence microscopy. BES: Bovine enamel slabs (control), B1a: 3 mol% yttria-stabilized tetragonal zirconia polycrystal surface; B2a: B1a with zirconium oxide (ZrO_2_) coating; B1b: B1a with zirconia-based composite coating; B2b: B1a with zirconia-based composite and zirconium oxide (ZrO_2_) coatings; * *p* ≤ 0.05, ** *p* ≤ 0.01.

The total bacterial count on the investigated implant materials after 2 h is presented by boxplots in Figure 2. The same figure also shows representative DAPI-stained microorganisms, which have adhered to the material surfaces after 2 h of incubation.

The differences between the materials were statistically significant. The lowest bacterial count of *E. faecalis* was detected on BES (*p* = 0.004) in comparison to the highest bacterial count on B1a. Three implant material surfaces, B1a (*p* = 0.047), B2a (*p* = 0.013) and B1b (*p* = 0.001) presented significantly higher bacterial count of *S. aureus* against the control BES. B2b showed no significant differences to the control (*p* = 0.065). However, significantly higher *C. albicans* count was detected on control (BES) surfaces compared to the groups B1a (*p* ≤ 0.001), B1b (*p* ≤ 0.001) and B2b (*p* ≤ 0.001). The surfaces belonging to group B2a harbored more *C. albicans* than B1a (*p* ≤ 0.001) and B1b (*p* ≤ 0.001).

Different implant materials with different surface characteristics were tested in this study (Table 2). The results indicated that the values of amplitude-, hybrid- and spatial parameters seem to play a major role with regard to initial adhesion. The smoother BES characterized by a lower surface roughness (Sa = 0.041 µm) behaved contrary to the rougher and micro-textured implant materials (Sa ≥ 0.2 µm) as far as the initial bacterial colonization is concerned. That is the reason why BES harbored less bacteria when compared to the tested material surfaces B1a, B1b, B2a and B2b. The fact that more *C. albicans* was recovered from B2a can be assumingly attributed to the anti-adhesive properties of zirconia-based composite coatings in B1a, B1b against fungi. Numerous studies have already highlighted the influence of surface topography on initial bacterial adhesion [20,21]. Al-Ahmad *et al.* [22] reported a positive correlation between high average surface roughness (Sa) and early microbial colonization after examining titanium and ceramic implants *in vivo*. This was confirmed in our study. According to the estimated bacterial counts the majority of microorganisms accumulated on the zirconia surfaces with high surface roughness (Sa ≥ 0.2 µm), while fewer microorganisms were detected on the smoothest surfaces of BES.

The amount of the attached microorganisms can be attributed to various parameters such as surface characteristics, bacterial concentration, exposure time and temperature [23,24]. High surface roughness provides bacteria with broad adhesion areas with irregularities to fit in avoiding shear forces and desorption due to maximum bacteria-surface contact during the initial adhesion phase [25,26]. In the presence of shear forces, especially *in situ*, surface characteristics such as amplitude parameters have a great impact on early bacterial adhesion [27]. Average surface roughness (Sa) lower than 0.2 µm did not influence the microbial adhesion, whereas Sa values higher than 0.2 µm were proposed to allow for higher initial bacterial adhesion in previous reports [28]. Sa correspondents to root mean square surface roughness (Sq), the deviation from the mean line/plane [29]. The ideal ratio of Ra:Rq is equal to 23/2π [30]. The Skewness (Rsk) representing the spatial variation in height differs between high, narrow hills with flat valleys (positive) and large, flat hills with narrow valleys (negative) [30].

The spatial roughness variations are defined by spatial parameters such as summit density (Sds) as well as developed area ratio (Sdr). Sds expresses the number of summits per unit area, while Sdr shows the percentage of additional area contributed by the surface texture [31]. Sdr correlates to the hydrophobicity of the surface. Okada *et al*. [32] reported that the inhibition of biofilm development resulted from hydrophilization of the substratum. The texture aspect ratio (Str) equals less than 1 and represents the relation of the shortest to the longest repeating surface pattern. All the aforementioned parameters describe the substratum contact area provided for microorganisms and play important roles in the analysis of initial microbial adhesion.

The selected implant materials as well as the tested bacterial strains seem to influence the bacterial accumulation on different substrata [33]. Rimondini *et al*. [34] compared the initial colonization of specific oral bacteria on Y-TZP *in vitro* and noted that *Streptococcus mutans* adhered significantly more to rectified Y-TZP than to titanium (Ti) disks. *Streptococcus sanguis* was mostly detected on Ti samples, whereas *Actinomyces* spp. and *Porphyromonas gingivalis* showed no differences.

To further prevent bacterial adhesion antimicrobial coatings on the implant surfaces were introduced [35,36]. Zirconium oxide-based composites in terms of implant surface modification have a low surface free energy and were suggested for coating zirconia to increase osteosynthesis by reducing bioinactivity [1]. The latter results from deficient intercommunication between Y-TZP substrata and cells [37]. Zirconia coatings have been reported to stimulate surface bioactivity due to their beneficial mechanical properties [38]. In the present report, no statistically significant differences were found between coated and uncoated specimens concerning *E. faecalis* and *S. aureus*. This is in accordance with a previous report, in which the antibacterial properties of silver-polysaccharide coatings on porous fiber-reinforced composites for bone implants were tested [39]. Only dense and porous samples differed statistically concerning the initial adhesion of *S. aureus* independent from the presence of coatings*.*

**Figure 3:**
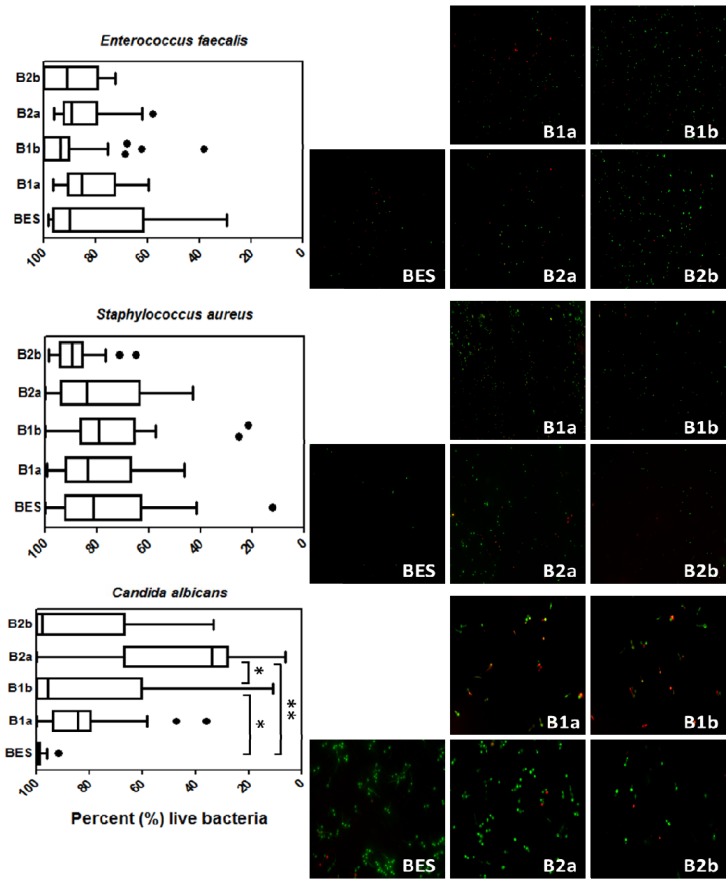
Boxplots exhibiting the viability of *Enterococcus faecalis*, *Staphylococcus aureus* and *Candida albicans* on the different implant material surfaces and the control after an exposure time of 2 h. The medians, whiskers, and Tukey outliers are displayed. The images on the right represent the fluorescence microscopic visualization of the adherent bacteria after the adoption of the live/dead staining. BES: Bovine enamel slabs (control); B1a: 3 mol% yttria-stabilized tetragonal zirconia polycrystal surface; B2a: B1a with zirconium oxide (ZrO_2_) coating; B1b: B1a with zirconia-based composite coating; B2b: B1a with zirconia-based composite and zirconium oxide (ZrO_2_) coatings; * *p* ≤ 0.05, ** *p* ≤ 0.01.

Figure 3 illustrates in box-plots and representative images the amount of vital adherent bacteria detected with the aid of live/dead staining on the investigated implant materials after 2 h.

The vitality differences between the surfaces were statistically significant only for *C. albicans*. The smallest amount of vital *C. albicans* strains (42.22%) was detected on B2a surfaces in comparison to the group BES (98.56%, *p* ≤ 0.001) with the maximum percentage of living microorganisms. The surfaces belonging to group B1b sheltered significantly more vital *C. albicans* (78.21%) than B2a (*p* ≤ 0.001) and significantly less than BES (*p* ≤ 0.001). The investigated implant material surfaces showed no significant discrepancies regarding the detection of *E. faecalis* and *S. aureus*. The majority of *E. faecalis* strains were found alive on all implant substrata: 80.56% (BES), 84.39% (B1a), 95.01% (B2a), 86.88% (B1b) and 92.57% (B2b). The vitality percentages of *S. aureus* were similar: 75.44% (BES), 78.64% (B1a), 74.42% (B2a), 78.90% (B1b) and 87.87% (B2b). All the aforementioned percentages represent the mean values of the recovered vital microorganisms.

It is already known that microbial adhesion to surfaces is a result of specific molecular interactions in the presence of physicochemical adhesion forces such as electrostatic and Lifshitz-Van der Waals forces [40]. Recently, it has been shown that microorganisms react variously to different adhesion forces originating from distinct surface materials [41]. Positive-charged substrata, for instance, quaternary ammonium-coated surfaces, develop strong attachment forces capable of eliminating negative-charged microorganisms (stained red by live/dead) due to stress deactivation [42]. The lethal positive charge surface density varies among the numerous bacterial species [43]. However, other substrata including ceramic materials interact differently with bacteria whose response progresses with increasing adhesion forces. These bacteria remain alive (stained green by live/dead) and are capable of producing extracellular polymeric substances (EPS) over time [8]. That explains why most *E. faecalis*, *S. aureus* and *C. albicans* strains were found vital (green fluorescence) when stained with Baclight live/dead dye in our report. The fact that less vital *C. albicans strains* were recovered from B2a when compared to B1b could be related to the lethal adhesion forces of zirconium oxide (ZrO_2_) coatings against fungi.

**Figure 4:**
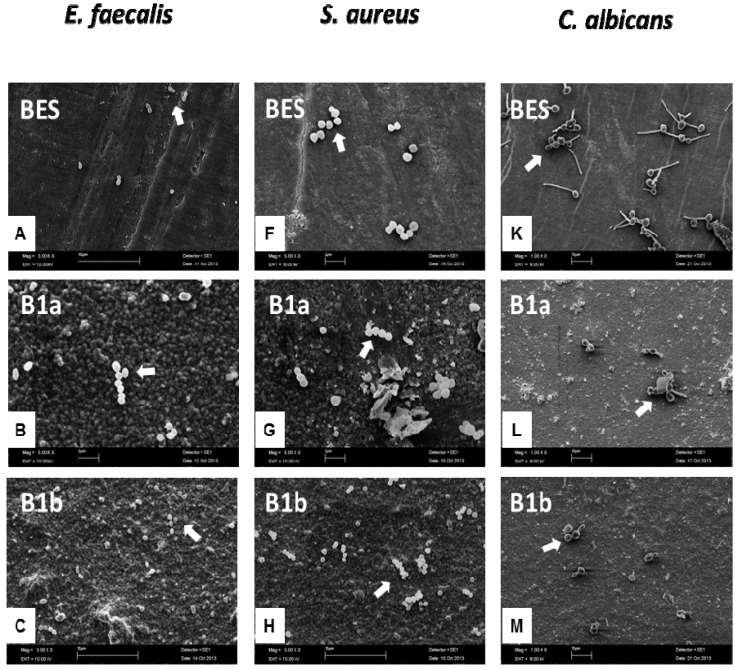

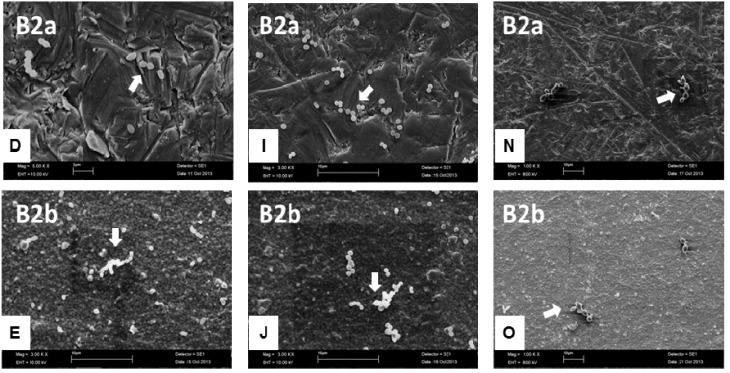
SEM images of *Enterococcus faecalis*, *Staphylococcus aureus* and *Candida albicans* on the different implant material surfaces as well as the control after an exposure time of 2 h. SEM magnification: 1000, 3000 and 5000 fold. The white arrows depict the microorganisms adhering to the implant surfaces. BES: Bovine enamel slabs (control); B1a: 3 mol% yttria-stabilized tetragonal zirconia polycrystal surface; B2a: B1a with zirconium oxide (ZrO_2_) coating; B1b: B1a with zirconia-based composite coating; and B2b: B1a with zirconia-based composite and zirconium oxide (ZrO2) coatings. Scale bar in B, D, F, G: 2 μm, scale bar in A, C, E, H–O: 10 μm.

The microbial adherence to all examined implant materials was visualized by SEM. Figure 4 demonstrates representative implant materials with adherent microorganisms.

The chains, pair-wise or single cocci of *E. faecalis and S. aureus* on all substrata are shown in Figure 4A–J. A significant increase in bacterial colonization could be shown for the implant groups B1a, B2a, B1b and B2b against the control BES surfaces. SEM images of candidal initial adhesion (Figure 4K–O) exhibited significantly more oval-shaped cells with characteristic cyto-plasmic evaginations on the BES than on the implant samples. The purpose of this report was to examine initial bacterial adhesion to different implant material surfaces *in vitro*. BES were suitable as a representative control group for the study of bacterial colonization on the implant surfaces due to their common physicochemical properties with human enamel [44]. In addition, homogenous BES can be easily gained, sterilized and are consistent in quality. Since the tested materials can be used for the fabrication of femoral heads for total hip replacements, oral and spinal implants, the microorganisms chosen can reside in the oral cavity and in other parts of the human body as well. The selection of the microorganisms was based on the fact that they represent characteristic organisms of persistent infections. The presence of microorganisms has been shown to be the main reason for implant failure [45]. *Enterococcus faecalis*, for example, is a Gram-positive strain, isolated from root-filled teeth with persistent secondary periapical lesions and endocarditis patients. *S. aureus* is an important nosocomial bacterium which can contaminate biomaterials. Moreover, *S. aureus* was isolated from periimplantitis patients and was shown to adhere to titanium implant materials [46]. *C. albicans* has been shown to constitute part of the oral biofilm. Furthermore, the present report aimed at testing the antimicrobial properties of the implant materials in terms of initial bacterial adhesion after 2 h. According to similar studies, this time period is considered satisfactory to confirm an anti-adhesive behavior of the surfaces. Their capability to allow for biofilm formation could be investigated in future studies.

To date, no study has examined the initial adherence of the selected microorganisms to these novel implant biomaterials. For studying initial bacterial adhesion *in vitro*, visualization methods such as SEM and DAPI staining were used. SEM was also appropriate for the observation of the biomaterial surfaces (Figure 1). Finally, live/dead staining added up some useful information about the vitality of the adherent microorganisms.

## 3. Experimental Section

### 3.1. Total Bacterial Count (DAPI)

Upon binding to double stranded DNA, the DAPI-molecule (4',6-diamino-2-phenylindole) stains DNA and therefore fluoresces intensely. The maximum fluorescence was observed at a wavelength of 461 nm. To count all adhered microorganisms, their microscopic analysis was performed according to Schwartz *et al*. [47].

For staining, three specimens of each examined implant material were inserted into the wells of multi-well plates (12-well plate; Greiner bio-one, Frickenhausen, Germany) and incubated with microorganisms for 2 h (37 °C, 5% CO_2_). Afterwards, the samples were covered with 2 mL DAPI (Merck, Darmstadt, Germany) solution (1 µg/mL in distilled water). After an incubation time of 10 min in a dark chamber, the DAPI solution was washed off with distilled water. The samples were then dried at room temperature and covered with Citifluor (Citifluor, Ltd, London, UK) on a slide. To quantify the total bacterial counts, the adherent microorganisms were analyzed using the Keyence BZ-9000 Fluorescence microscope (Keyence Germany; Neu-Isenburg, Germany). The average fluorescence intensity of the detected microorganisms was determined with the aid of “BZ image analysis application”, an image acquisition software utilizing single cell tracing algorithm. The number of cells observed in 10 randomized microscopic ocular grid fields per sample was counted. The area of ocular grid (0.043 mm^2^) allowed estimating the number of cells per cm^2^. The experiment was conducted in duplicates and repeated twice. Representative images were shot for illustration of the results.

### 3.2. Live/Dead Staining

For the main experiments, SYTO^®^ 9 stain and propidium iodide (PI) (Live/Dead^®^ BacLight™ Bacterial Viability Kit, Life Technologies GmbH, Darmstadt, Germany) were selected [48]. The fluorescent agent was dissolved in a 0.9% saline (NaCl) solution to a final concentration of 0.1 nmol∙mL^−1^. For staining, three specimens of each examined implant material were inserted into the wells of multi-well plates (12-well plate; Greiner bio-one, Frickenhausen, Germany). After incubation with microorganisms for 2 h (37 °C, 5% CO_2_), the samples were stained with 2 mL SYTO^®^ 9/PI in 0.9% NaCl in a dark chamber for 10 min at room temperature and mounted with superglue (Loctite 401, Loctite Deutschland GmbH, Munich, Germany) on slides. Microscopic analysis using the Keyence BZ-9000 Fluorescence microscope (Keyence Germany; Neu-Isenburg, Germany) followed directly afterwards. The measurement of the average fluorescence intensity of the detected microorganisms was conducted with the aid of “BZ image analysis application”, an image acquisition software based on single cell tracing algorithms. The number of microorganisms per cm^2^ detected in 10 randomized microscopic ocular grid fields (0.043 mm^2^ each) per sample was determined. The measurement was performed in duplicates and repeated twice. Representative images were acquired for demonstration of the outcomes.

### 3.3. Scanning Electron Microscopy (SEM)

After fixation in 8% formaldehyde for 3 days at 4 °C, the specimens were dehydrated in an ascending ethanol series (30%, 50%, 70%, 80%, 90% once each and twice in 100% for 1 h). Then critical point drying (Critical Point Dryer CPD 030; Bal-Tec, Wallruf, Germany) was conducted, the samples were sputter-coated with gold/palladium in an SCD 050 coater (Bal-Tec, Wallruf, Germany) and finally examined by a Zeiss Leo 435 VP scanning electron microscope (Leo Electron Microscopy Ltd Cooperation Zeiss Leica, Cambridge, UK) at 10 kV.

### 3.4. Statistical Analysis

For descriptive demonstration of the data, boxplots were generated and graphically displayed, stratified by bacterial count/cm^2^ and material. The presence of significant differences among the different measured parameters was analyzed by one way ANOVA and Tukey test. The significance level was *p* ≤ 0.05. Statistical analysis was performed by IBM SPSS statistics 19.0.

## 4. Conclusions

The results of this *in vitro* study confirmed the impact of various surface characteristics on the initial bacterial adhesion. Compared to bovine enamel slabs (BES) the coated and uncoated zirconia biomaterial substrata exhibited no anti-adhesive properties when contaminated with *E. faecalis*, *S. aureus* and *C. albicans* for 2 h. A further improvement of the zirconia-based surface parameters towards lower surface roughness values can be recommended to reduce the risk of bacterial adhesion leading to implant failure.
